# Wharton’s jelly-derived mesenchymal stem cells ameliorate high altitude-induced heart injury by promoting type 2 macrophage polarization via COX2-PGE2 pathway

**DOI:** 10.3389/fimmu.2025.1538046

**Published:** 2025-06-06

**Authors:** Wenhong Zhang, Li Zhao, Jing Cui, Yan Zhang, Dongtao Li, Zhibo Hong, Jiamin Liu, Shan Wang, Ningkun Zhang, Yang Li, Yu Chen

**Affiliations:** ^1^ The Fifth School of Clinical Medicine, Anhui Medical University, Hefei, Anhui, China; ^2^ Department of Cardiology, The Sixth Medical Center of Chinese People’s Liberation Army (PLA) General Hospital, Beijing, China; ^3^ Beijing Anzhen Hospital, Capital Medical University, Key Laboratory of Remodeling-related Cardiovascular Diseases, Ministry of Education, Beijing Collaborative Innovation Centre for Cardiovascular Disorders, Beijing, China; ^4^ Beijing Institute of Heart, Lung and Blood Vessel Disease, Beijing, China

**Keywords:** Wharton’s jelly-derived mesenchymal stem cells, high-altitude-induced heart injury, hypobaric hypoxia, macrophage polarization, inflammation

## Abstract

**Background:**

Chronic high-altitude hypobaric hypoxia leads to high-altitude heart disease and heart failure. Recent research has indicated that WJMSCs (Wharton’s jelly-derived mesenchymal stem cells, WJMSCs) can alleviate ischemic myocardial injury and improve cardiac dysfunction, and macrophage polarization may have been involved. However, few studies have focused on the cardioprotective effects of WJMSCs against HAHI (high-altitude-induced heart injury, HAHI). Here, our research focused on how WJMSCs regulate macrophage polarization impacted myocardial repair in HAHI.

**Methods:**

C57/BL6J mice were fed for 28 days at a hypobaric chamber that had a comparable altitude of 6000 m, and WJMSCs were injected intravenously before HH (hypobaric hypoxia, HH) exposure. To assess cardiac function, echocardiography was carried out. Blood and heart tissue were collected for subsequent analysis. We simulated anoxic environment *in vitro* by inducing BMDMs (bone marrow-derived macrophages, BMDMs) with 1% O_2_, and employed co-culture system to investigate how WJMSCs affect macrophage polarization.

**Results:**

Abnormal myocardial fibrosis and cardiomyocyte apoptosis, cardiac inflammation and dysfunction were exhibited in the Chronic HAHI mouse model. WJMSCs infusion maintained the cardiac structure and function in HAHI mice. Furthermore, WJMSCs infusion was effective in elevating the M2 macrophages proportion and decreasing inflammation in the heart. *In vitro* studies revealed that hypoxia stimulation elevated the ratio of M1 macrophages in comparison to those in the Control group and coculturing with WJMSCs encouraged the shift of M1 to M2 macrophages. Surprisingly, the anti-inflammatory effects of WJMSCs on M2 polarization were negated with pretreatment of a COX2 (Cyclooxygenase-2, COX2) inhibitor, which could be reversed with PGE2 (prostaglandin E2, PGE2) addition.

**Conclusions:**

In conclusions, our findings indicated that WJMSCs infusions may enhance M2 macrophage polarization through the COX2-PGE2 pathway, and therefore safeguard against cardiac damage in HAHI mice.

## 1 Introduction

High-altitude regions are featured by low temperature and HH (1). At high altitudes, HH exposure can have harmful effects on many organs, particularly the heart because of its high oxygen consumption. The heart is susceptible to hypoxic stress, and prolonged exposure to HH in high-altitude areas could result in impairment of myocardial structure and function (2). Hypoxia plays a significant role in the development of CVDs (cardiovascular diseases, CVDs). When normoxia is not restored, there is an increased production of proinflammatory cytokines, such as IL-1β, IL-6, and TNF-α. This condition also triggers a metabolic shift from fatty acid oxidation to glycolysis, potentially leading to cardiac hypertrophy, myocarditis, and ultimately heart failure. (3, 4). Regrettably, current treatments for HAHI, such as acetazolamide, dexamethasone and nifedipine, have significant toxic side effects on the human body (5, 6). Therefore, there is an immediate demand for safer and more effective therapeutic approaches. MSCs (Mesenchymal stem cells, MSCs), derived from sources such as umbilical cord, bone marrow, adipose tissue, amniotic membranes, and dental pulp, possess the capability to differentiate into osteoblasts, adipocytes, and chondroblasts under standard *in vitro* conditions (7). The role of MSCs in tissue repair and regeneration is facilitated by paracrine effects and highly plastic immune regulation (8, 9). They also have anti-fibrotic properties because they reduce the deposition of extracellular matrix (ECM) and fibrosis-associated factors in tissues (10–12), and protect against pathological damage and cardiomyocyte apoptosis (13). WJMSCs from the umbilical cord isolated from Wharton’s jelly have a higher capacity for proliferation, stronger immunomodulatory effects, less ethical concerns, and are safer than MSCs derived from other sources (14). Owing to these properties, WJMSCs have become an ideal source for the treatment of various diseases, including HAHI. This study is designed to further explore the therapeutic effect and mechanism of WJMSCs in HAHI.

Chronic inflammation regulated by macrophages is a crucial factor in the aggravation or alleviation of HAHI. The roles of macrophage phenotypes vary in inflammatory diseases. M1 macrophages release pro-inflammatory cytokines and chemokines, inflaming and causing cardiac damage. M2 macrophages, another phenotype of macrophage, assist in the healing of damaged tissue by producing anti-inflammatory cytokines (15). Therefore, the inflammatory response and cardiac damage could be improved by increasing the number or proportion of M2 macrophages.

More recently, it has been shown that the activation of macrophages by HH causes cardiac inflammation in both ventricles, resulting in hypersecretion of iNOS (inducible nitric oxide synthase, iNOS) and cytokines (IL-1β, IL-18 and C-Reactive Proteins) (16). Adverse cardiac remodeling is accelerated by the pro-inflammatory responses, which activates and maintains the fibrotic cascade, ultimately leading to heart failure and sudden cardiac death (17, 18). Furthermore, it had been reported that infarction repair was connected to the transformation of M1 macrophages into M2 macrophages, and heart failure and adverse ventricular remodeling are more likely to occur in infarcted hearts with delayed M2 polarization (19, 20). Interestingly, both *in vivo* and *in vitro*, MSCs have been proven to have a powerful effect on M2 macrophage polarization (21–23). Moreover, it has been reported that the immunomodulatory effects of MSCs are closely related to the secretion of PGE2. MSCs can induce macrophage M2 polarization through the secretion of PGE2, thereby reducing inflammation and promoting tissue repair (24, 25). However, little is known about hypoxia-mediated inflammation in the non-ischemic/non-infarcted heart, and effects of WJMSCs in treating HAHI are unclear, and whether the COX2-PGE2 signaling pathway is enhanced in WJMSCs under hypoxia needs further research. Specifically, whether this therapy can reduce cardiomyocyte apoptosis, inflammation, and HH-related fibrosis through promoting M2 macrophage polarization via COX2-PGE2 pathway deserves further investigation.

In this research, HAHI mouse model and *in vitro* experiments were utilized to examine the effectiveness of WJMSCs in repairing cardiac injury caused by HH and polarizing M2 macrophages. Our aim was to elucidate the protective effects and underlying mechanisms of WJMSCs on HAHI in mice, potentially paving the way for novel therapeutic strategies in high-altitude-induced cardiac complications.

## 2 Materials and methods

### 2.1 Cell culture

#### 2.1.1 Mouse bone marrow-derived macrophages isolation and culture

BMDMs were isolated following established protocols (26). The bone marrow was centrifuged at 500g for 5 minutes, and the cells were resuspended in complete medium (DMEM/F12, 10% fetal bovine serum, 1% penicillin-streptomycin, 20ng/mL macrophage colony-stimulating factor). Subsequently, the cells were cultured for an additional 6 days. The complete medium was refreshed every 72 hours. After a period of 7 days, we assessed the purity of macrophages using flow cytometry. This was accomplished by staining the cells with CD11b-PE (BioLegend) and F4/80-FITC (BioLegend) antibodies.

#### 2.1.2 WJMSCs culture

WJMSCs were isolated as described previously (27); Approval for the study design was obtained from the Ethics Review Committee of the Sixth Medical Center of the People’s Liberation Army in China. Additionally, written informed consent forms were secured from participants. WJMSCs were cultivated with serum-free mesenchymal basal medium (Yocon, China, NC0106) under controlled conditions of 37°C and 5% CO2 incubator. WJMSCs were passaged 4 to 7 times, and identified by positive staining with antibodies against surface CD73-FITC (BioLegend), CD90-FITC (BioLegend) and CD105-FITC (BioLegend) and negative staining with antibodies against CD34-FITC (BioLegend), CD45-FITC (BioLegend), HLA-DR-FITC (BioLegend) by flow cytometry. The target cells we chose were in the FSC-Height and SSC-Height quadrant to separate cell debris. Please refer to our previous publication to obtain more details (28).

### 2.2 Animals and treatments

The Ethics Review Committee of the Sixth Medical Center of the People’s Liberation Army in China has granted approval for our animal experiments in accordance with the animal management regulations established by the Ministry of Health of China. Forty-eight healthy SPF male C57BL/6J (23-25g) were obtained from the animal laboratory center of GENE LINE BIOSCIENCE (permit number JLHK-20230325-01) in China. Mice were confined to a constrained environment at 22 ± 2°C, with humidity levels ranging from 45% to 55%, and a 12h-12h light/dark schedule. Mice were permitted to engage in activities without any limitations and had free access to food and water. Four groups of mice were randomly distributed as follows (n=12): Control; NN (normobaric normoxia, NN) + WJMSCs; HH + NS (neutral saline, NS); HH + WJMSCs. For the Chronic HAHI mouse model, mice were maintained for 28 days at an equivalent altitude of 6000m (450-500hpa) in a hypobaric chamber (Yantai Hao Te Oxygen Chamber). In the WJMSCs treatment experiment, 100ul WJMSCs (5 × 10^5^ cells) were injected into the tail vein of the mice before HH exposure. Mice that were under normobaric normoxia environment and administered an equivalent volume of NS served as the Control group (NN+NS).

### 2.3 General state of mice

Mice were measured for their body weight over a 28-day period.

### 2.4 Echocardiography

A high-resolution ultrasound imaging system (VINNO 6, Vinno Corporation, Suzhou, China) was employed for the echocardiographic assessment. Briefly, mice were anaesthetized with isoflurane (3% for induction and 2% for maintenance, vol./vol.). To record the systolic and diastolic motion profiles of the left and right ventricles, M-mode echocardiography was carried out. Measurements and records were taken for the LVEF (left ventricular ejection fraction, LVEF), LVFS (left ventricular fractional shortening, LVFS), LVIDD (left ventricular internal diastolic dimension, LVIDD), PAPV (pulmonary artery peak velocity, PAPV), and RVID (right ventricular internal diameter, RVID).

### 2.5 Tissue and blood harvesting

Once the echocardiography measurements were complete, the animals were sacrificed by cervical dislocation (n=12). Freshly isolated hearts were immediately snap-frozen in liquid nitrogen (-196°C) and stored at -80°C until further use. Blood samples were collected for ELISA analysis.

### 2.6 Western blot analysis

RV (right ventricle, RV) tissues and cells were used to extract proteins and to be homogenized. Homogenates were utilized for protein extraction, which included the proteins listed below. Collagen I, collagen III, α-SMA (Alpha-smooth muscle actin, α-SMA), BAX (B-cell lymphoma 2-associated X protein, BAX), Bcl2 (B-cell lymphoma-2, Bcl2), cleaved-caspase3, CD206, CD86, IL-10, TNF-α and COX2 were analysis by Western blot. The total protein concentration was determined by BCA protein assay (PC0020, Solarbio, Beijing, China). Equal amounts of proteins (20μg/sample) were loaded onto a gradient gel and subsequently transferred to a membrane (ISEQ00010, biosharp, Beijing, China). The membrane was then blocked with 5% milk in PBST and incubated overnight with antibodies at 4°C. The antibody was diluted as follows: anti-collagen I (ab260043, 1:1000, Abcam), anti-collagen III (ab184993, 1:1000, Abcam), anti-α-SMA (ab124964, 1:1000 dilution, Abcam), anti-BAX (cat#141707, 1:1000, proteintech), anti-Bcl2 (cat#226593-1-AP, 1:3000, proteintech), anti-cleaved-caspase3 (cat#AF7022, 1:2000, proteintech), anti-CD206 (ab64693, 1:1000, Abcam), anti-CD86 (13395-1-AP, 1:4000, proteintech), anti-TNF-α (ab307164, 1:1000, Abcam), anti-IL-10 (ab310329, 1:1000, Abcam), and anti-COX2 (ab179800, 1:1000, Abcam). To account for variations in protein expression, β-actin (cat#20536-1-AP, 1:6000, proteintech) was employed for internal calibration as a control. Analysis of the protein bands was performed using Image-Pro Plus software.

### 2.7 Histological staining

After endpoint echocardiography measurements, three mice of each group were sacrificed. Hearts were fixed in 4% paraformaldehyde for 72 hours following their collection. After fixation, the hearts underwent dehydration and were embedded in paraffin. Subsequently, they were sliced into sections with a thickness of 5 μm. To assess the degree of fibrosis in the left and right ventricles, Masson’s trichrome staining was employed. The Image-Pro Plus software (v6.0, Media Cybernetics Inc, Bethesda, MD) software was adopted to calculate collagen volume fraction (CVF): CVF = the blue collagen fiber area in the visual field**/**the total area of the myocardial tissue in the visual field. Inflammatory cell infiltration was assessed using hematoxylin-eosin (HE) staining. M1 macrophage polarization was determined by immunofluorescence of F4/80 and CD86, and M2 macrophage polarization by F4/80 and CD206.

### 2.8 Immunofluorescent staining

For immunostaining, the heart tissue sections were incubated overnight at 4 °C with primary antibodies, including anti-CD86 (BM4121, 1:500, Boster), anti-CD206 (ab64693, 1:1000, Abcam), and anti-F4/80 (GB11027, 1:500, Servicebio). The following day, the sections were washed with PBS three times before being incubated with a secondary antibody (cat#5220-0336, 1:400, Seracare) at room temperature for one hour and TSA amplification (cat#11065, AAT Bioquest; G1231, Servicebio) were applied to label F4/80 as well as CD86 and CD206, respectively, followed by antigen retrieval. Finally, the sections were counterstained with DAPI and mounted on a coverslip. The slides were observed under a microscope (Mshot).

### 2.9 Enzyme-linked immune sorbent assay

The concentrations of IL-6, TNF-α, IL-10 and PGE2 in the cell culture and serum were measured using commercially available ELISA kits (MULTI SCIENCES). The optical density at 450 nm was assessed using a Multiskan MK3 Microplate Reader (Thermo).

### 2.10 *In vitro* experiments

BMDMs were obtained as previously described (26). Then, to identify the effect of hypoxia on the polarization of macrophages, stimulation was conducted by AnaeroPack-CO_2_ and AnaeroPouch-Anaero bags (MITSUBISHI GAS CHEMICAL CO., Inc., Japan) (95% N_2_ and 5% CO_2_), coculturing with WJMSCs in a 6-well culture plate at a ratio of 1 WJMSC: 2 macrophages for 12 h in a Transwell system (Corning), with WJMSCs located in the upper chamber at 1.5 ml and BMDMs located in the lower chamber at 2 ml. After 12h coculturing, supernatants and cells were collected for further analysis. Two wells were used for each group. The cells were separated into groups as follows: (1) Control group, (2) hypoxia group, (3) hypoxia + WJMSCs, (4) hypoxia + WJMSCs + COX2 inhibitor (20 μM, MedChemExpress), and (5) hypoxia + WJMSCs + COX2 inhibitor + prostaglandin E2 (PGE2; 0.2μM, MedChemExpress). WJMSCs in groups 4 and 5 were administered with a COX2 inhibitor for a duration of 2 hours prior to their co-culture with macrophages, either in the presence or absence of PGE2.

### 2.11 Flow cytometry analysis

Flow cytometry was done in accordance with standard protocols. Fluorochrome-tagged monoclonal antibodies from BioLegend were added to the cells and incubated for 30 minutes at 4°C, such as anti-mouse CD11b PE (cat#101207, 0.2mg/ml, BioLegend), anti-mouse F4/80 FITC (cat#123107,0.5mg/ml, BioLegend), anti-mouse CD86 PE (cat#105007, 0.2mg/ml, BioLegend), and anti-mouse CD206 APC (cat#141707, 0.2mg/ml, BioLegend). Cell populations were gated as follows: M1 macrophages (F4/80^+^CD86^+^) and M2 macrophages (F4/80^+^CD206^+^). We conducted flow cytometry using a DxFlex flow cytometer (BeckManCoulTer) and analyzed the data with FlowJo software (FlowJo, LLC).

### 2.12 Statistical analyses

We used GraphPad Prism 9.5.0 software to analyze the data. All values for statistical analysis were presented as the means ± standard deviation. One-way or two-way analysis of variance (ANOVA) was employed to assess the mean differences among distinct groups. Statistical significance was determined with a *p*-value threshold set at 0.05.

## 3 Result

### 3.1 Characterization of WJMSCs and BMDMs

**Figure 1:**
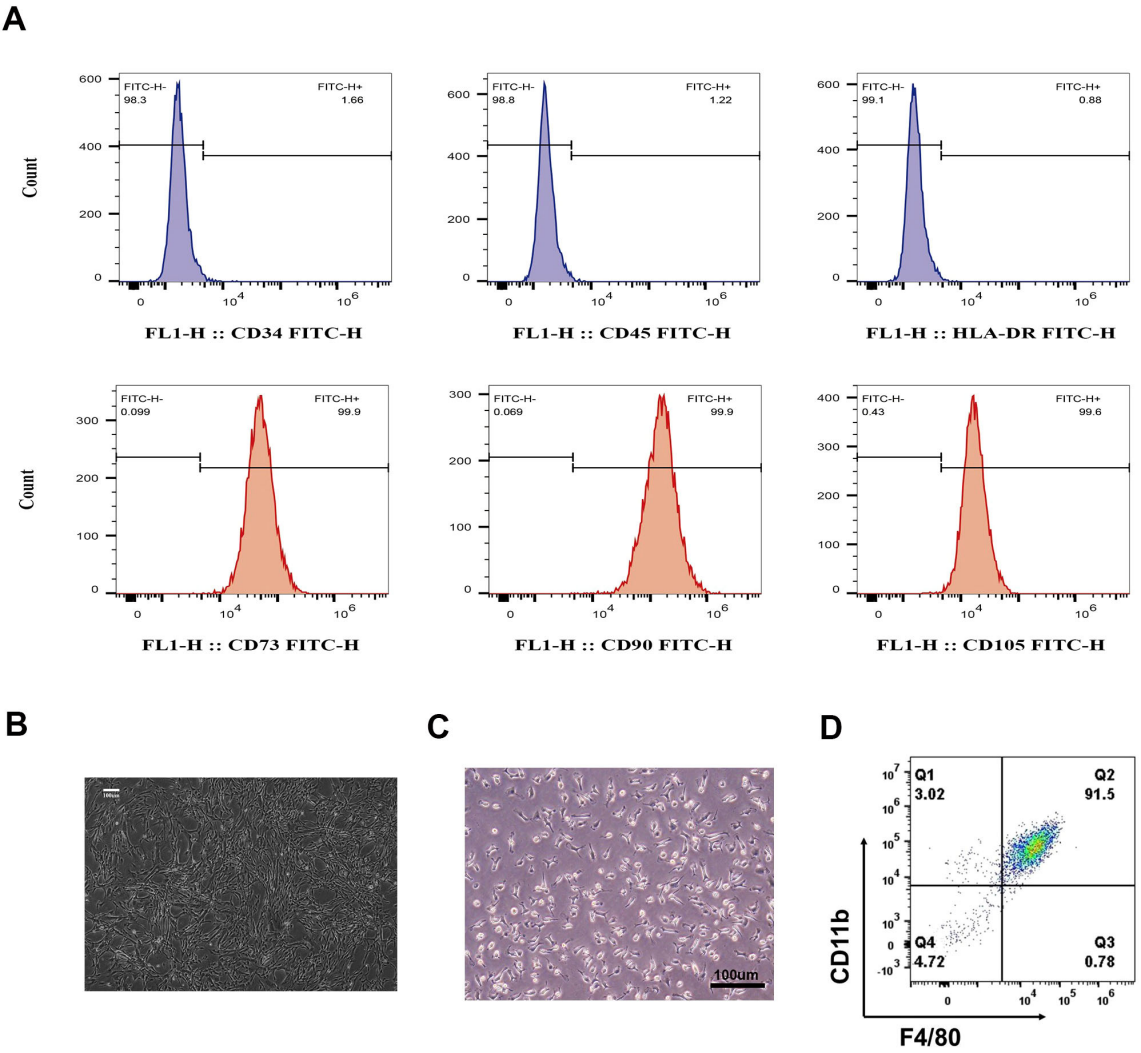
Identification of WJMSCs and BMDMs. **(A)** Cultivated Passage 4 to 7 WJMSCs stained positive for CD73-FITC, CD90-FITC, and CD105-FITC, but negative for CD34-FITC, CD45-FITC, and HLA-DR-FITC via flow cytometry. The target cells we chose were in the FSC-Height and SSC-Height quadrant to separate cell debris. **(B)** WJMSCs morphology was observed via light microscopy. **(C)** BMDMs morphology was observed via light microscopy, and **(D)** more than 90% of the cultured macrophages stained positive for CD11b and F4/80 via flow cytometry. Scale bar: 100 μm.

After four passages, WJMSCs were identified according to their morphology and phenotype. Flow cytometry showed that more than 90% of the cells were positive for CD73, CD90, and CD105, but negative for CD34, CD45, and HLA-DR (**Figure 1A**). WJMSCs showed a typical spindle-shaped morphology, grew in clusters, and formed a vortex-like monolayer (**Figure 1B**).

The characteristics of BMDMs were also determined by their morphology and phenotype. As shown in **Figure 1C**, the macrophages were characterized by their high refractivity, large size, and irregular shape. Flow cytometry data revealed that the cell population was highly pure (> 90%) and tested positive for specific surface antibodies F4/80 and CD11b (**Figure 1D**), indicating that the macrophages derived from mouse bone marrow were successfully cultured.

### 3.2 Effect of WJMSCs on the general state of mice

**Figure 2:**
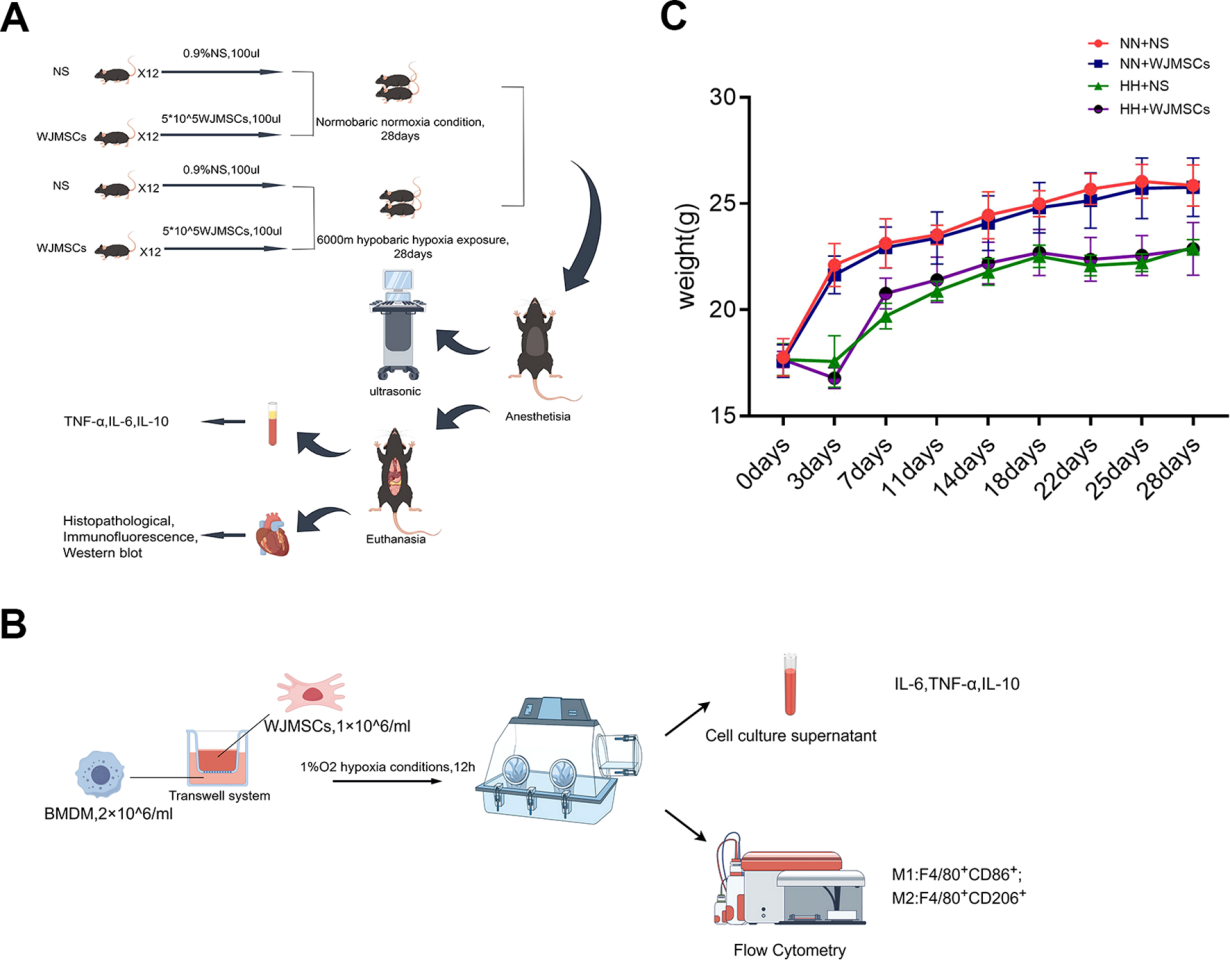
Schematic illustration of the animals and cells experimental protocols of the study and body weight curve. **(A, B)** Schematic illustration of the animals and cells experimental protocols of the study. **(C)** Body weight at 0d, 3d, 7d, 11d, 14d, 18d, 22d, 25d and 28d. Values are expressed as mean ± SD (n = 6).

Schematic illustration of the *in vivo* and *in vitro* studies is presented in (**Figures 2A, B**). In the experiment, mice’s general health was observed and compared. Changes of mice body weight in each group were exhibited in the line chart (**Figure 2C**). There was no discrepancy in body weight between the groups at day 0. The control and NN + WJMSCs groups exhibited normal weight gain. However, HH + NS and HH + WJMSCs groups experienced significant weight loss after HH exposure at day 3. In contrast, the body weight of the mice in other groups increased rapidly, indicating that HH exposure significantly inhibited the growth and development of mice. No significant difference in body weight was observed between HH + NS group and HH + WJMSCs group.

### 3.3 WJMSCs improved cardiac function

**Figure 3:**
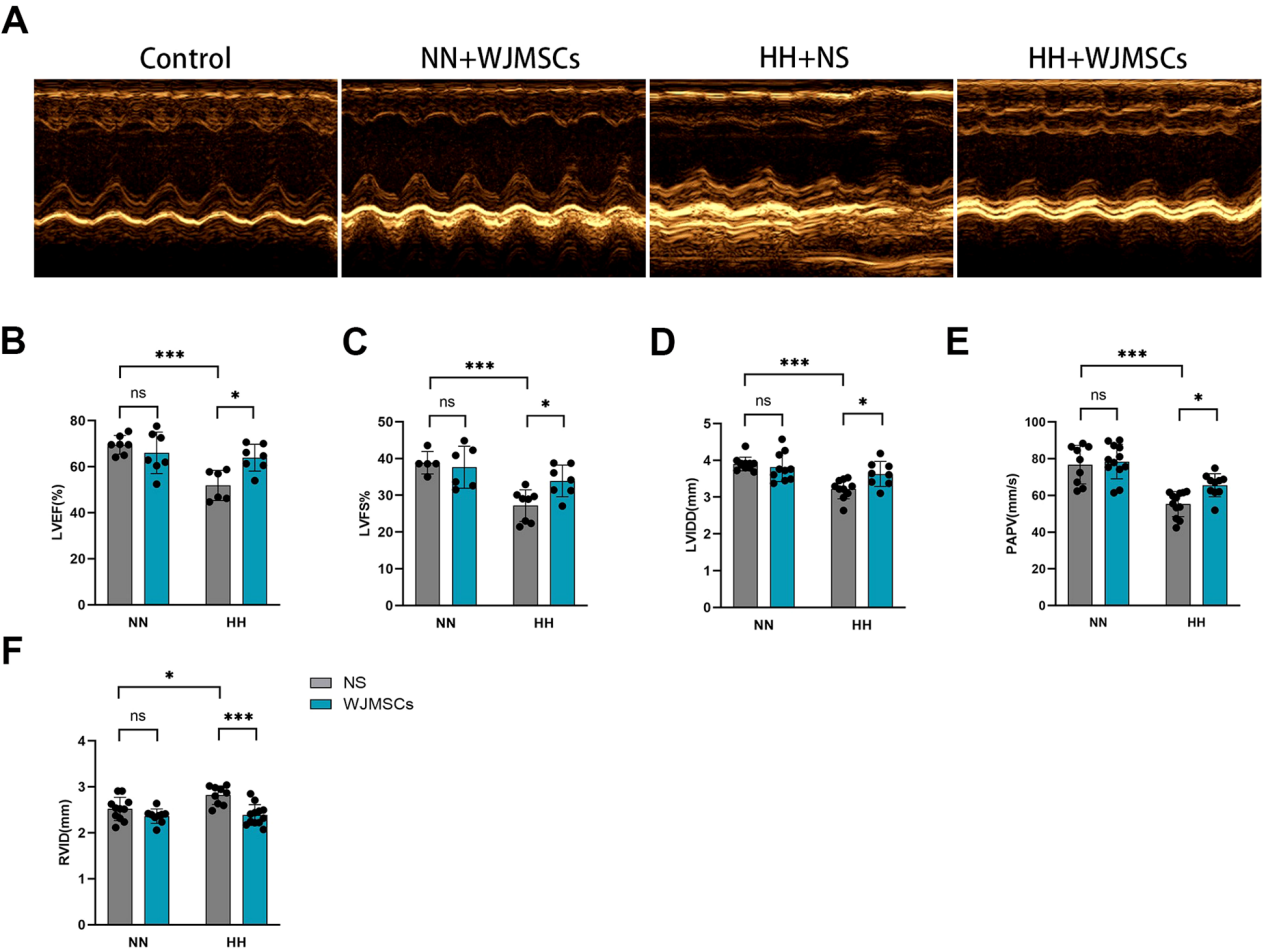
Effects of WJMSCs on cardiac function in HH-exposed mouse model. **(A)** Representative M-mode echocardiogram of each group is performed after 28-day HH exposure. **(B–F)** The changes in LVEF, LVFS, LVIDD, PAPV, and RVID of each group on days 28 after HH exposure. All data were analyzed by two-way analysis of variance (ANOVA). Data was expressed as mean ± SD (n = 5-12). *P < 0.05, ***P < 0.001. ns, not significant; LVEF, left ventricular ejection fraction; LVFS, left ventricular fractional shortening; LVIDD, left ventricular internal diastolic dimension; PAPV, pulmonary artery peak velocity; RVID, right ventricular internal diameter; NN, normobaric normoxia; HH, hypobaric hypoxia; NS, neutral saline; WJMSCs, Wharton’s jelly-derived mesenchymal stem cells.

We investigated the effect of WJMSCs on cardiac function. We found that mice exposed to HH exhibited a marked reduction in LVEF and LVFS when compared to the control group, which indicated HH exposure impaired left ventricular systolic function (*P*<0.05), while WJMSCs treatment could significantly increase LVEF and LVFS, compared to the HH exposure mice (*P*<0.05) (**Figures 3A, B, C**). HH exposure decreased LVIDD and PAPV, while increased RVID, indicating a higher right heart pressure and pulmonary artery resistance (*P*<0.05). WJMSCs infusion mitigated these HH-induced changes (*P*<0.05) (**Figures 3D, E, F**).

### 3.4 WJMSCs attenuated cardiac remodeling

**Figure 4:**
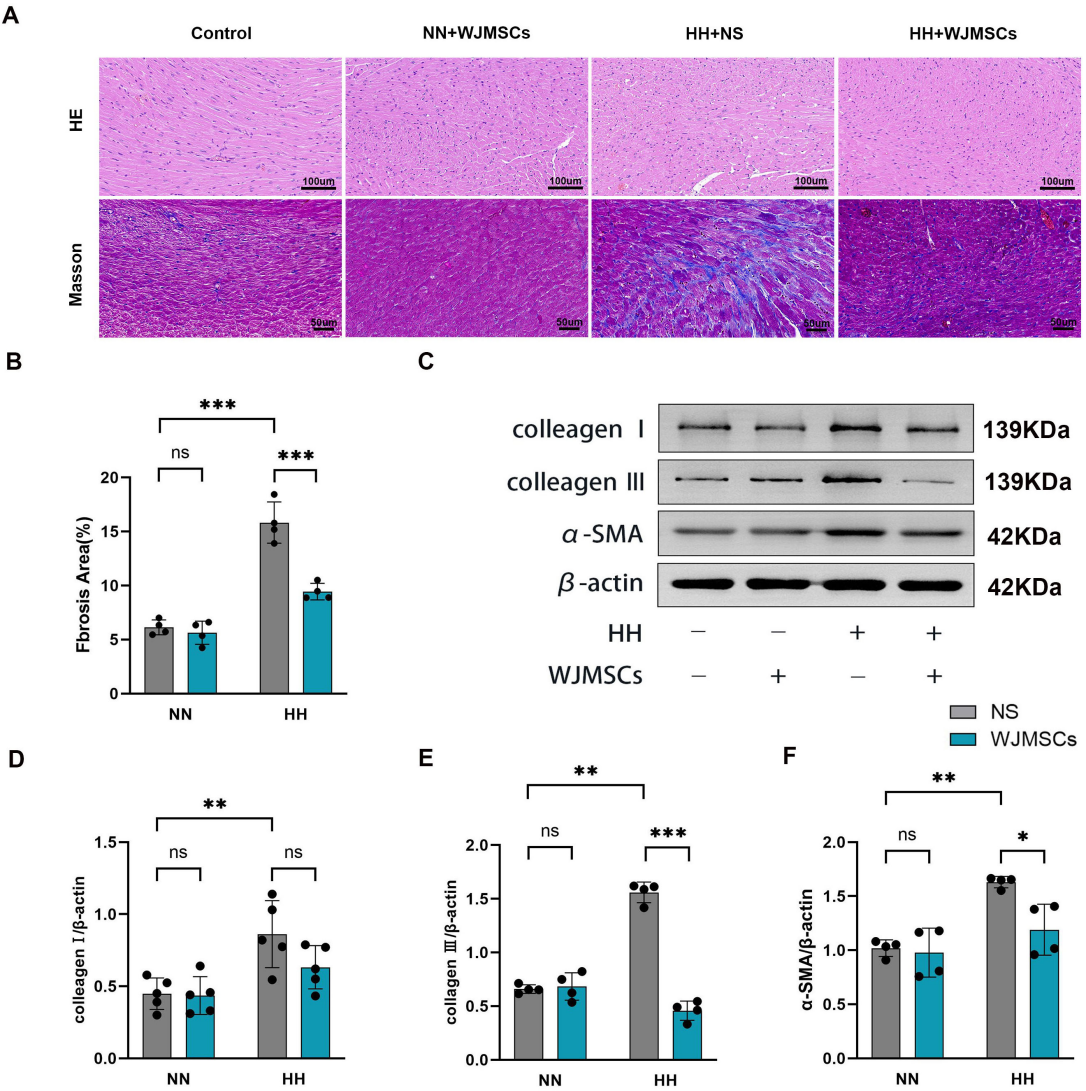
Effects of WJMSCs on RV histology in HH-exposed mouse. **(A)** Representative photomicrograph of the morphological changes of RV tissue in HH-exposed mouse by HE staining (40×) and Masson’s trichrome staining (40×). **(B)** Statistical analysis of fibrosis of RV tissue. **(C)** Analysis of collagen I, collagen III and α-SMA expression level, detected by western blot. **(D–F)** Statistical analysis of collagen I, collagen III and α-SMA expression level of RV tissue. All data were analyzed by two-way analysis of variance (ANOVA). Data was expressed as mean ± SD (n = 4-5). *p < 0.05, **p < 0.01, ***p < 0.001. ns, not significant; α-SMA, α-smooth muscle actin; NN, normobaric normoxia; HH, hypobaric hypoxia; NS, neutral saline; WJMSCs, Wharton’s jelly-derived mesenchymal stem cells.

According to HE staining, there was a disturbance in the structure of the myocardium, with inflammatory cells infiltrating, space widening, rupture, dissolution, and microbleeds in the HH + NS group, while WJMSCs treatments significantly improved the pathological changes of heart tissue. For further study, the levels of fibrosis in these mice were evaluated by Masson’s trichrome staining. It was discovered that HH exposure significantly increased the area of myocardial fibrosis, which was reduced by WJMSCs infusion, exhibited by means of gross images (**Figure 4A**), and by quantification (*P*<0.05) (**Figure 4B**). To further confirm these findings, we performed western blot using anti-collagen III, anti-collagen I, and anti-α-SMA antibodies (**Figure 4C**). The results were consistent, that HH causes severe heart collagen deposition, showing a marked increase in fibrotic protein expression (collagen III, collagen I, and α-SMA) in the HH + NS group (*P*<0.05). These pathological changes caused by HH were ameliorated by WJMSCs pre-treatment (**Figures 4D, E, F**).

### 3.5 Effects of WJMSCs on apoptosis in the heart of mice following HH exposure

**Figure 5:**
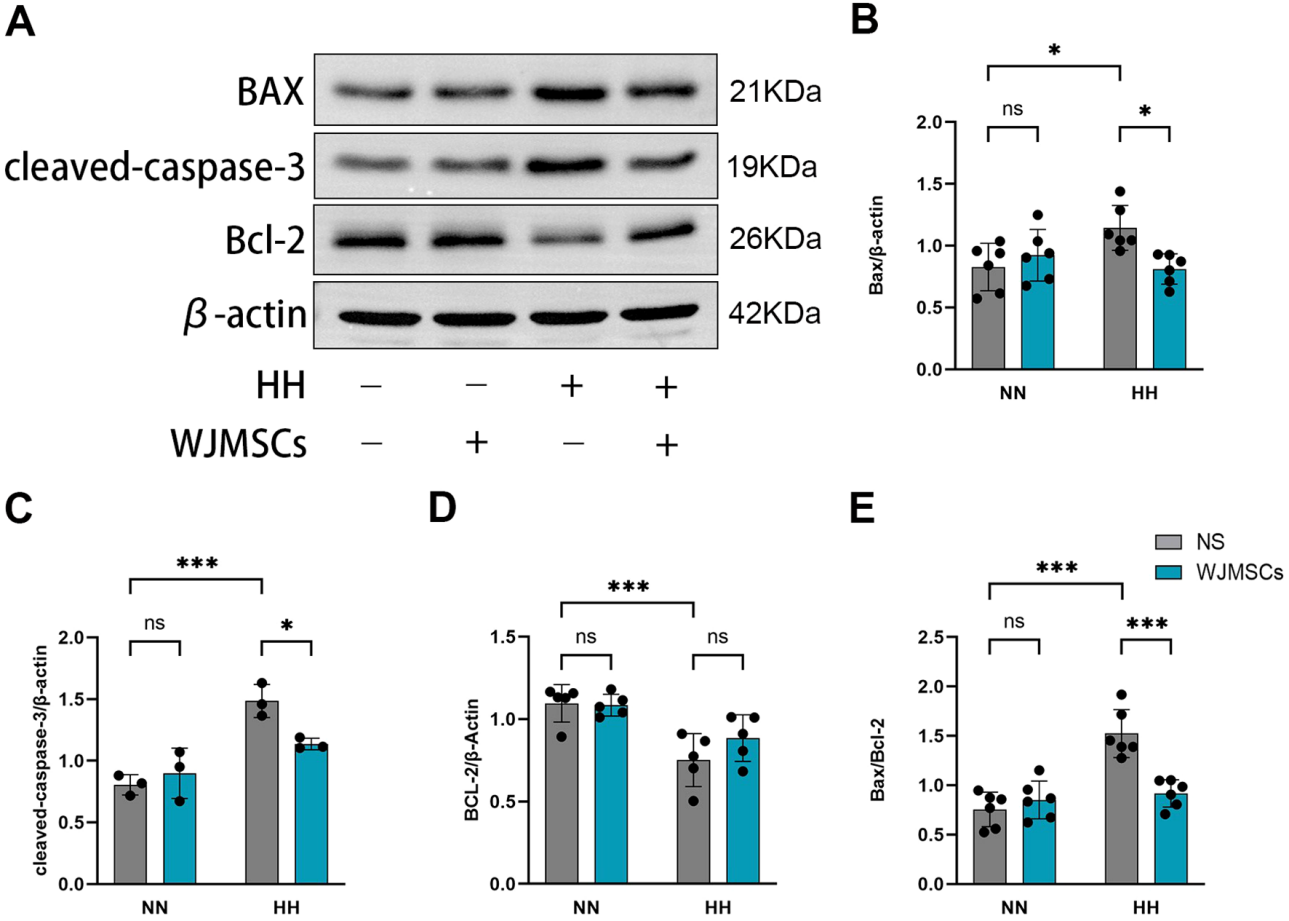
Effects of WJMSCs on the apoptosis-related proteins expression in HAHI mice by Western blot. **(A)** Western blot analysis of the expression of apoptosis-related proteins in hearts. **(B–E)** Quantitative results of relative expressions of apoptosis-related proteins. All data were analyzed by two-way analysis of variance (ANOVA). All values were expressed as mean ± SD (n = 3-6). *p < 0.05, ***p < 0.001. ns, not significant; Bax, B-cell lymphoma 2-associated X protein; Bcl-2, B-cell lymphoma 2. NN, normobaric normoxia; HH, hypobaric hypoxia; NS, neutral saline. WJMSCs, Wharton’s jelly-derived mesenchymal stem cells.

As revealed in **Figure 5A**, HH exposure resulted in an increase in the levels of Bax, cleaved caspase-3, and the Bax/Bcl-2 ratio, while simultaneously decreasing Bcl-2 expression in comparison to the Control group (*P*<0.05). By contrast, WJMSCs administrations effectively reversed these changes in cardiac tissues under HH environment (**Figures 5B, C, D, E**).

### 3.6 WJMSCs inhibits hypobaric hypoxia-induced inflammatory responses

**Figure 6:**
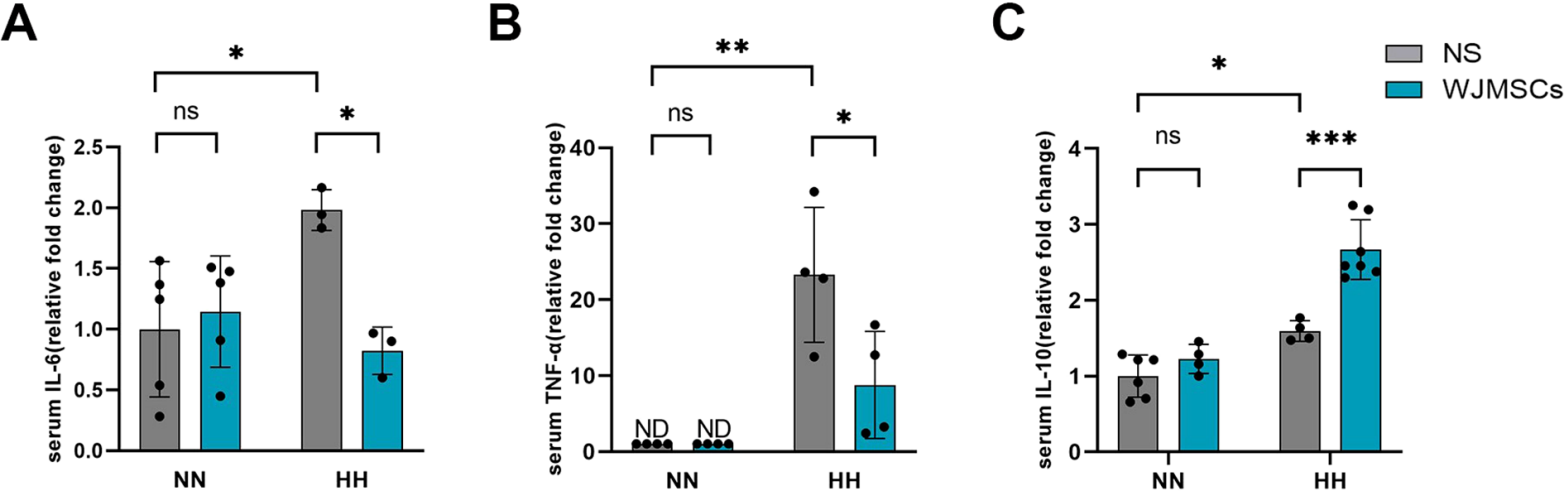
Effects of WJMSCs on cardiac inflammatory cytokine levels in HAHI mice. Levels of **(A)** IL-6, **(B)** TNF-α and **(C)** IL-10 in serum. All data were analyzed by two-way analysis of variance (ANOVA). Values are expressed as mean ± SD (n = 3-7). *p < 0.05, **p < 0.01, ***p < 0.001. ns, not significant; HH, hypobaric hypoxia; NN, normobaric normoxia; IL-6, interleukin 6; TNF-α, tumor necrosis factor α. IL-10, interleukin 10. NS, neutral saline; WJMSCs, Wharton’s jelly-derived mesenchymal stem cells; ND, not detected.

In comparison to the Control group, the HH + NS group showed significantly higher serum concentrations of IL-6 and TNF-α, indicating that proinflammatory factors were involved in HAHI (*P*<0.05). WJMSCs pre-treatment markedly inhibited HH-induced pro-inflammatory factor release (*P*<0.05) (**Figures 6A, B**), and significantly increased the IL-10 levels compared with those in the HH + NS group (*P*<0.05) (**Figure 6C**). These results indicate that WJMSCs pre-treatment may prevent HAHI by suppressing inflammation.

### 3.7 The effect of WJMSCs on promoting M2 macrophage polarization in HH-induced mice hearts

**Figure 7:**
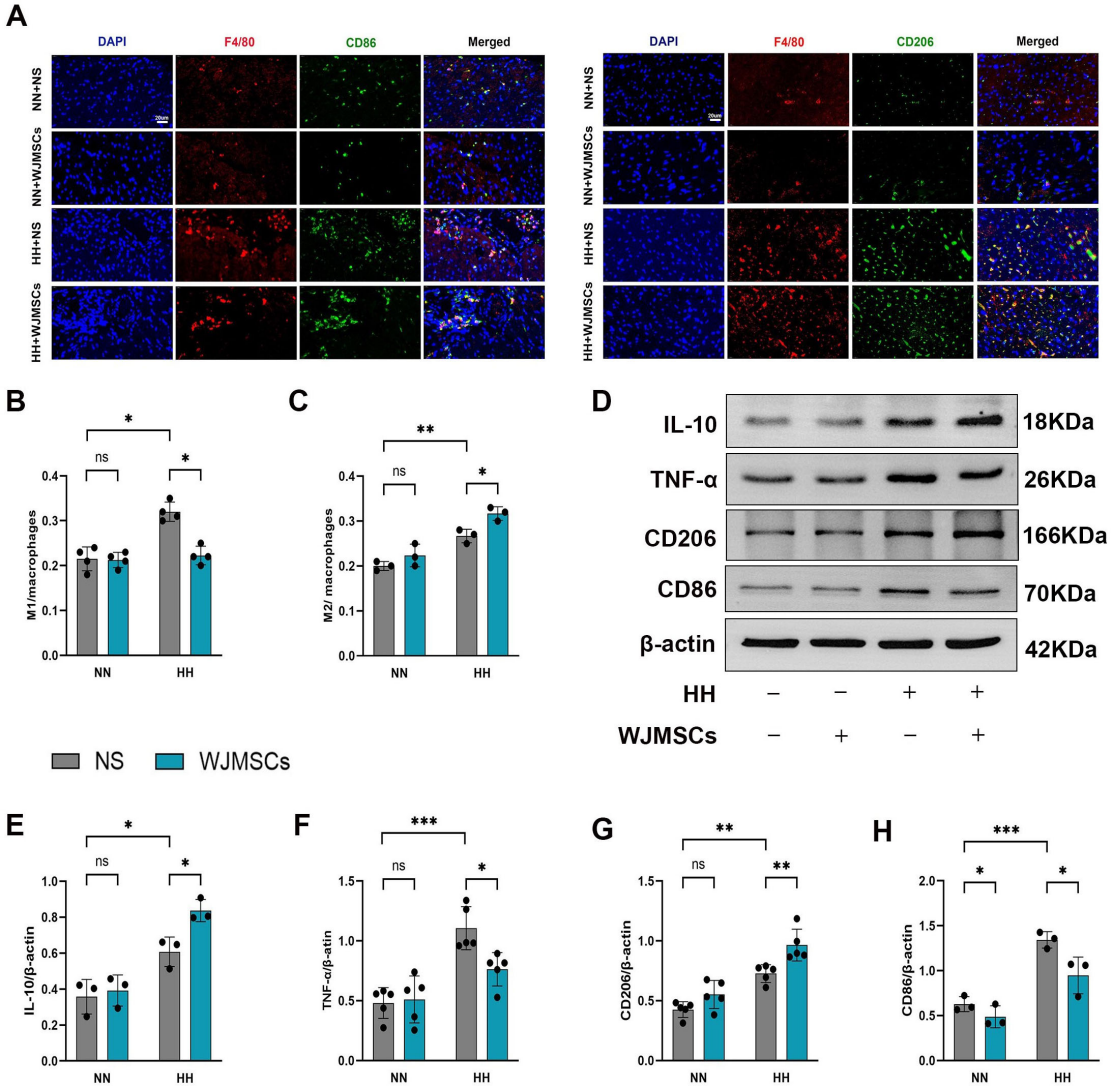
WJMSCs inhibited pro-inflammatory macrophages and promoted anti-inflammatory macrophages in RV. **(A)** Representative images showed the CD86^+^ pro-inflammatory macrophages (green) and CD206^+^ anti-inflammatory macrophages (green) in each group. The nuclei were stained with DAPI (blue), and macrophages were stained with F4/80 (red). Scale bar, 20μm. **(B–C)** The percentage of CD86^+^ or CD206^+^ cells were calculated by Image-Pro Plus. **(D–H)** The protein level of CD86, CD206, TNF-α and IL-10 were measured by western blot, and quantification of the relative expression in the bands in different groups was calculated by Image-Pro Plus. All data were analyzed by two-way analysis of variance (ANOVA). Values are expressed as mean ± SD (n = 3-5). *p < 0.05, **p < 0.01, ***p < 0.001. ns, not significant; HH, hypobaric hypoxia; NN, normobaric normoxia; IL-10, interleukin 10; TNF-α, tumor necrosis factor α. NS, neutral saline; WJMSCs, Wharton’s jelly-derived mesenchymal stem cells.

To explore whether WJMSCs treatment affected the aggregation and polarization of macrophages, we labeled total macrophages with F4/80, M1 macrophages with CD86 and M2 macrophages with CD206 in the right ventricle by immunofluorescence. The results indicated that both F4/80^+^CD86^+^ (M1) and F4/80^+^CD206^+^ (M2) macrophages exhibited an increase in the HH + NS group compared to the Control group, with a notable rise in M1 (*P* < 0.05). Interestingly, in comparison to the HH + NS group, treatment with WJMSCs significantly reduced M1 macrophage infiltration while promoting an increase in M2 macrophages in the right ventricle (*P*<0.05) (**Figures 7A, B, C**). Next, we detected the protein markers of M1 and M2 macrophage in the right ventricle with western blot (**Figure 7D**). In the HH + NS group, the levels of protein expression for CD206 and CD86 were significantly elevated compared to the Control group (*P*<0.05), with CD86 showing a particularly notable increase. In contrast, treatment with WJMSCs resulted in a significant rise in CD206 expression while concurrently reducing the levels of CD86 when compared to the HH + NS group (*P*<0.05) (**Figures 7G, H**). In addition, our study revealed that HH exposure led to elevated levels of TNF-α and IL-10 proteins in the right ventricle (*P*<0.05). However, when treated with WJMSCs, TNF-α got a significant decrease, and IL-10 significantly increased. (*P*<0.05) (**Figures 7E, F**). According to these findings, WJMSCs not only decreased macrophage infiltration but also elevated the proportion of M2 macrophages in the hearts of HAHI mice.

### 3.8 WJMSCs promoted M2 macrophage polarization and alleviated inflammation under the hypoxic environment *in vitro*

**Figure 8:**
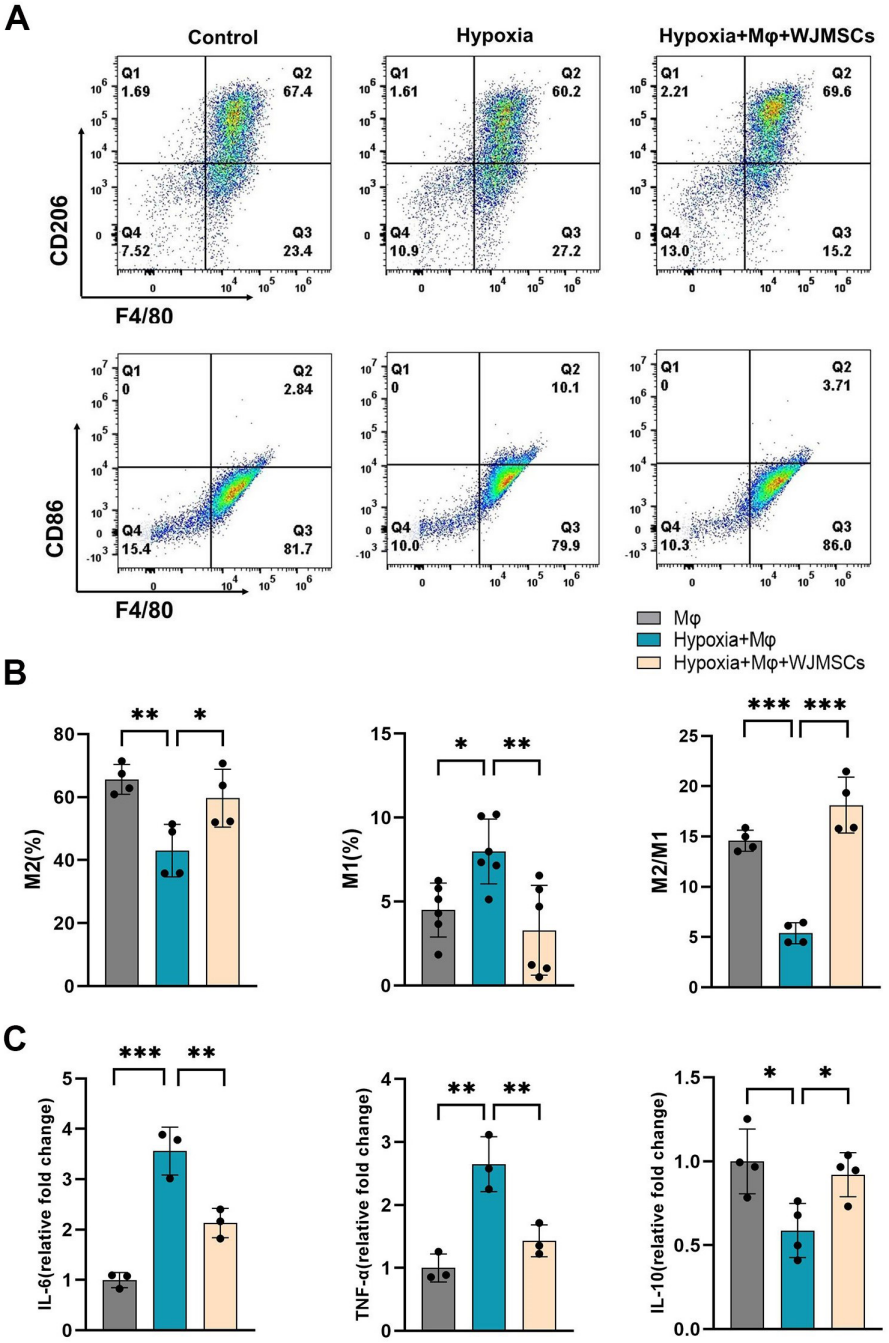
WJMSCs promoted M2 macrophage polarization and anti-inflammatory effects under hypoxic environment *in vitro*. **(A)** Mice BMDMs were separated and divided into the normal, hypoxia, and hypoxia + WJMSCs groups. After treatment, macrophages from the different groups were collected, and the M1 percentages, M2 percentages and the ratios of M2 to M1 phenotypes were detected by flow cytometry. **(B)** The data were analyzed using Graphpad prism 9.5.0 software. **(C)** The IL-6, TNF-α and IL-10 concentrations in the supernatants were determined by ELISA. All data were analyzed by one-way analysis of variance (ANOVA). Data was expressed as mean ± SD (n = 3-6). *p < 0.05, **p < 0.01, ***p < 0.001. IL-6, interleukin 6; IL-10, interleukin 10; TNF-α, tumor necrosis factor α. NS, normal saline; WJMSCs, Wharton’s jelly-derived mesenchymal stem cells.

To further validate that WJMSCs alleviated cardiac chronic inflammation by activating M2 macrophage, we designed a cell co-culture system under the hypoxic environment *in vitro*. BMDM cells were co-cultured with WJMSCs in a transwell system for 12 h, and analysis was done to determine the ratio of different macrophage subtypes. As shown in **Figures 8A, B**, hypoxia stimulation elevated the ratio of M1 and declined the ratio of M2 in comparison to those in the Control group (*P*<0.05). Furthermore, it was found that coculturing with WJMSCs promoted the shift of M1 to M2 macrophages, which was indicated by an increasing proportion of M2 macrophage, whereas the opposite occurred for M1 macrophages (*P*<0.05). To confirm these findings, the inflammatory cytokines were analyzed. The results indicated a notable increase in IL-6 and TNF-α levels, alongside a significant reduction in IL-10 within the supernatant of the Hypoxia group (*P*<0.05) (**Figure 8C**). However, WJMSCs cocultured with macrophages reversed these changes by inhibiting IL-6 and TNF-α secretion, while IL-10 was promoted (*P*<0.05). These data indicated that hypoxia activates the M1 macrophage phenotype, resulting in an elevated production of pro-inflammatory cytokines such as IL-6 and TNF-α. Conversely, WJMSCs are capable of polarizing macrophages toward the M2 phenotype and promoting the release of IL-10.

### 3.9 WJMSCs regulated M2 polarization via the COX2−PGE2 pathway

**Figure 9:**
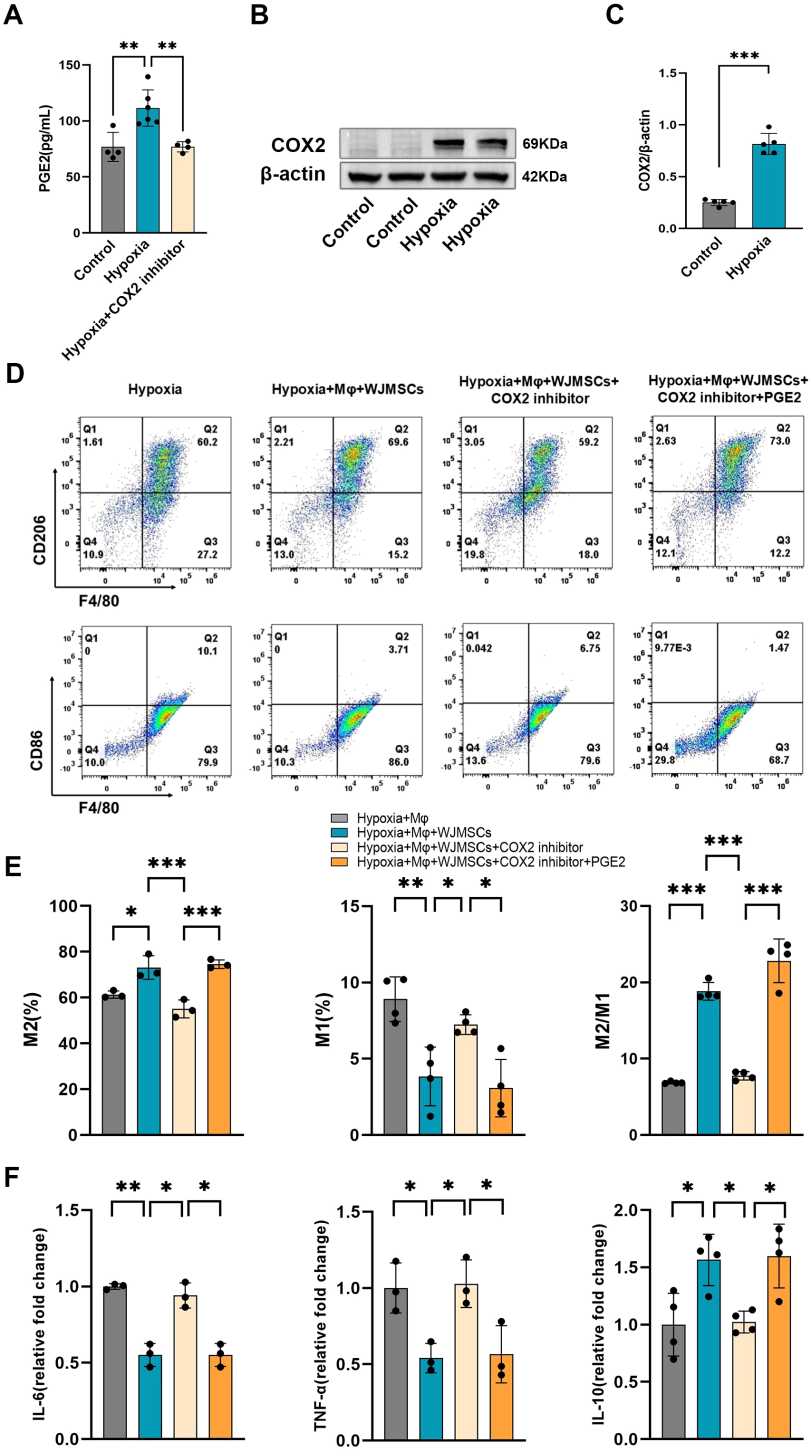
WJMSCs promoted M2 macrophage polarization and anti-inflammatory effects under the hypoxic environment via the COX2-PGE2 pathway. **(A)** WJMSCs were incubated in control, Hypoxia or Hypoxia + COX2 inhibitor media, and the PGE2 concentration in the supernatant was detected by ELISA. **(B)** Western blot analysis of the expression of COX2 of WJMSCs in the control and the hypoxia group. **(C)** Statistical analysis of COX2 expression level. **(D-E)** The hypoxia-induced macrophages were cocultured with WJMSCs pretreated with or without COX2 inhibitor in the presence or absence of exogenous PGE2. After treatment, macrophages were collected, and the M1 and M2 phenotypes were detected by flow cytometry. **(F)** The IL-6, TNF-α and IL-10 concentrations in the supernatants were detected by ELISA. All data were analyzed by one-way analysis of variance (ANOVA). Data was expressed as mean ± SD (n = 3-6). *p < 0.05, **p < 0.01, ***p < 0.001. IL-6, interleukin 6; IL-10, interleukin 10; TNF-α, tumor necrosis factor α. NS, normal saline; WJMSCs, Wharton’s jelly-derived mesenchymal stem cells.

The immunoregulatory effects of MSCs have been demonstrated by previous studies to be related to PGE2 (9), and the secretion of PGE2 by MSCs was significantly decreased by COX2 inhibition (25). Our results also showed that the concentration of PGE2 in the culture supernatant of WJMSCs was increased under hypoxia. In addition, inhibition of COX2 significantly reduced the expression of PGE2 by WJMSCs (**Figure 9A**). In mutual corroboration, hypoxia stimulated WJMSCs to highly express COX2 protein, the key enzyme for PGE2 synthesis (**Figures 9B, C**). Therefore, we proposed that PGE2 might be responsible for the polarization of M2 macrophages induced by WJMSCs. To confirm this, we designed the experiment *in vitro*, dividing the cells into five groups: Control; Hypoxia; Hypoxia + WJMSCs; Hypoxia + WJMSCs + COX2-inhibitor; Hypoxia + WJMSCs + COX2-inhibitor + PGE2, and then assessed the macrophage polarization. As indicated by the research, M1 macrophages in the hypoxia group experienced a significant rise while M2 macrophages decreased markedly (*P*<0.05). When cocultured with WJMSCs, the polarization of M1 and M2 macrophages was dramatically reversed (*P*<0.05). However, inhibiting COX2 resulted in an observable increase in M1 macrophages, while a significant decrease in M2 macrophages in comparison to the Hypoxia + WJMSCs group (*P*<0.05). Following the addition exogenous of PGE2 into the COX2-inhibited group, the polarizations of M1 and M2 macrophage were drastically reversed (*P*<0.05) (**Figures 9D, E**). Additionally, the IL-6 and TNF-α levels went up sharply, while the IL-10 significantly fell in Hypoxia group (*P*<0.05) (**Figure 9F**). Coculturing with WJMSCs reduced the IL-6 and TNF-α and increased IL-10 (*P*<0.05). Moreover, the COX2-inhibited group experienced a significant elevation in IL-6 and TNF-α, while a drastic decrease in IL-10 (*P*<0.05). IL-6 and TNF-α levels were inhibited by exogenous PGE2, while IL-10 levels were increased (*P*<0.05). As indicated in the results, PGE2 produced by WJMSCs could activate M2 macrophages, which in turn could reduce inflammation caused by hypoxia.

## 4 Discussion

The onset and advancement of HAHI is closely associated with high-altitude exposure, which poses a significant threat to both physical and mental health (29). However, effective treatment strategies for HAHI remain elusive. Current pharmacological interventions, such as acetazolamide, dexamethasone and nifedipine, while beneficial in maintaining vascular endothelial function and improving myocardial ischemia, carry notable side effects (5, 6). Umbilical cord-derived WJMSCs possess superior proliferative capacity, stronger immunomodulatory effects, less ethical concerns, and greater safety than MSCs derived from other sources (14, 30). Owing to these properties, WJMSCs have become a promising source for the treatment of HAHI.

Although there is an amount of information available on right ventricular hypertrophy caused by pressure overload from pulmonary hypertension, hypoxia is known to activate multiple molecular pathways that may play a role in both the hypertrophic response and the dilation of the RV. Research conducted under hypoxic conditions has demonstrated a significant involvement of oxidative stress (31), kinase activation (32), and inflammatory processes (33) in hypoxia-induced adaptive hypertrophy and interventricular remodeling. Moreover, macrophage polarization is involved in the progression of numerous heart diseases, such as atherosclerosis, myocardial infarction, diabetic cardiomyopathy and myocarditis (34). However, there is still no investigation about the connection between macrophage polarization and HAHI. In this study, our findings demonstrates that macrophage polarization is a crucial factor in HAHI.

Microenvironments differ in their polarization of macrophages to different phenotypes and functions (35, 36). M1 macrophages are known for producing pro-inflammatory cytokines, such as IL-6 and TNF-α, along with chemotactic factors that intensify inflammation and myocardial damage (37–39). Conversely, M2 macrophages are the ones who perform the repair and reduction of inflammation (40) by secreting reparative factors like TGFβ1 (transforming growth factor-β1, TGFβ1), IL-10, and VEGFA (vascular endothelial growth factor A, VEGFA). Previous studies have indicated that the injured myocardium is the target of circulating monocytes, which substitute resident macrophages (mainly M2), and then to be converted into M1 macrophages, which subsequently worsen myocardial injury (41, 42). Our research demonstrated that both M1 and M2 were present in the HAHI model, with a predominance of the M1 phenotype. However, WJMSCs pre-administration markedly reduced M1 markers while upregulating M2 markers, suggesting a shift towards a more reparative phenotype. We measured the expression of CD86, CD206, TNF-α and IL-10, and further demonstrated that WJMSCs ameliorated HAHI by shifting M1 macrophages toward M2 phenotype. Similar to HAHI, AMI (acute myocardial infarction, AMI) is a prototypical sterile injury. According to previous research, MSCs transplantation can be beneficial for cardiac repair in animal models or patients suffering from AMI (43, 44).

It has been observed that the transition from M1 to M2 macrophages is associated with the repair of damaged cardiac tissue. Moreover, delayed polarization of M2 macrophages in infarcted hearts increases the likelihood of adverse ventricular remodeling and the onset of heart failure (20). Thus, macrophage polarization is thought to be a potential therapeutic target for AMI. Moreover, it had been confirmed that MSCs could efficiently transform M1 macrophages into M2 phenotypes both *in vivo* and *in vitro*.

Chronic inflammation, mediated by macrophages, plays a pivotal role in HAHI progression. Accumulating evidence revealed that inflammation is another factor that leads to right ventricular remodeling (45). Previous studies reported that inflammatory factors, particularly IL-6 and TNF-α, are key factors in initiating cascade inflammatory responses (46). IL-6 and TNF-α can accelerate the secretion of a large amount of collagen and myocardial fibrosis in myocardial tissue, and finally damage myocardial function (34, 47). TGFβ1 corresponds to a smaller infarct size and enhances angiogenesis in infarcted hearts (48). IL-10, a cytokine that is primarily expressed in activated T lymphocytes and stimulated monocytes, has powerful anti-inflammatory properties and prevents excessive inflammatory responses (49). IL-10 inhibits proinflammatory cytokine and chemokine production in endotoxin-stimulated macrophages, mitigating inflammatory responses (50). Furthermore, IL-10 may have a significant impact on ECM remodeling by encouraging the production of Tissue Inhibitor of Metalloproteinases (TIMP)-1, which helps stabilize the matrix (49). Myocardial collagen deposition, especially collagen I and collagen III, is considered the primary factor in cardiac fibrosis (51). α-SMA is one of markers for myofibroblasts. Abnormal persistence of the myofibroblast is a hallmark of fibrotic diseases (47). Our results align with these observations, demonstrating that RV remodeling and fibrosis were exhibited in HAHI mice. Additionally, the results also revealed that HH exposure led to a rise in the concentration of pro-inflammatory factors (IL-6, TNF-α) both in plasma and myocardial tissue. However, both RV fibrosis and plasma inflammation were inhibited with the administration of WJMSCs. In addition, the level of IL-10 in both blood and heart tissue was higher after WJMSCs pretreatment. Therefore, WJMSCs therapy can reduce inflammation, and inhibit fibrosis by promoting macrophage M2 polarization, thus promoting the repair of HAHI.

Relevant research have demonstrated that HH exposure increases cardiac apoptosis in both rat and mouse models (52), and increased IL-6 and TNF-α can also induce the decline of myocardial cell vitality and apoptosis (53, 54). Thus, we further examined the apoptosis-regulating proteins Bax, cleaved-caspase-3, and Bcl2. Our findings revealed a significant increase in Bax and cleaved-caspase-3 levels, alongside a reduction in Bcl2. Notably, these effects were counteracted by the prior administration of WJMSCs. The downregulation of proteins associated with apoptosis and fibrosis in the heart tissues indicates that WJMSCs can inhibit HH-induced myocardial apoptosis in the heart.

Furthermore, we investigated the potential mechanism for WJMSCs to regulate macrophage polarization *in vitro*. As demonstrated by previous studies, the regulation of MSC-modulated inflammation in sepsis is closely associated with PGE2 (24, 25). Whether the COX2-PGE2 signaling pathway is enhanced in WJMSCs under hypoxia has not been reported. The results of our study showed that the expression of PGE2 in the culture supernatant of hypoxic WJMSCs was significantly increased, and the key enzyme of PGE2 synthesis, COX2, was significantly expressed. To further verify this pathway, we designed a hypoxic WJMSCs co-culture experiment with BMDMs *in vitro* and flow cytometry was used. Additionally, it has been reported that M1 type macrophages polarization was enhanced through culturing BMDMs under hypoxic condition (55, 56), which was similar to our results. Moreover, hypoxia caused M1 macrophages to polarize and release proinflammatory cytokines. Consistent with previous research, our findings indicated that coculturing with WJMSCs boosted the M2 macrophages proportion and the release of anti-inflammatory cytokines *in vitro*. To investigate the role of PGE2 in the M2 polarization induced by WJMSCs, we employed a key inhibitor of PGE2 synthesis, specifically a COX2 inhibitor (57). *In vitro* hypoxia group, the production of pro-inflammatory cytokines increased and M1 macrophage polarization could not be reversed when macrophages were cocultured with WJMSCs pretreated with a COX2 inhibitor. However, the effects were revived by the addition of exogenous PGE2 to the coculture system. Based on these studies, it is suggested that the COX2-PGE2 pathway might be responsible for WJMSCs-induced M2 macrophage activation in HAHI.

There are several limitations that need to be noted. First, the simulated environment was HH, but the actual plateau environment was more complicated, for instance, apart from HH, there were also low temperatures, strong ultraviolet light, and various other factors. Second, the mechanistic verification at the animal level was not sufficient in our study. Finally, in our *in vitro* studies, we utilized a non-contact co-culture system for WJMSCs and BMDMs, but in myocardial tissue, there may be multiple types of cell-to-cell communication or signals exchange, which need further investigation.

## 5 Conclusion

To summarize, our results demonstrated that WJMSCs infusion preserved cardiac structure and function in HAHI mouse model. Moreover, WJMSCs exert their beneficial effects by alleviating cardiomyocyte apoptosis, reducing inflammation, and inhibiting HH-related fibrosis, partly through COX2-PGE2 pathway, which promotes M2 macrophages polarization in the hearts. These discoveries hold the promise of an innovative and efficient approach to treating high-altitude-related heart disease.

## Data Availability

The original contributions presented in the study are included in the article. Further inquiries can be directed to the corresponding author.
